# Whole-tumor histogram analysis of non-Gaussian diffusion MRI models combined with clinicopathological indicators for assessing lymph node metastasis in endometrial cancer

**DOI:** 10.3389/fmed.2026.1959334

**Published:** 2026-10-22

**Authors:** Yaqi Zang, Hongxia Wang, Wenpeng Ge, Huijia Yin, Lizhi Xie, Ruijing Qin, Wangyi Liu, Dongming Han

**Affiliations:** 1 Department of Magnetic Resonance, The First Affiliated Hospital of Henan Medical University, Xinxiang, Henan, China; 2 MR Research China, GE Healthcare, Beijing, China; 3 First Teaching Hospital of Tianjin University of Traditional Chinese Medicine, Tianjin, China

**Keywords:** diffusion kurtosis imaging, endometrial cancer, histogram analysis, lymph node metastasis, non-Gaussian diffusion MRI

## Abstract

**Objectives:**

To investigate whether whole-tumor histogram features derived from five non-Gaussian diffusion models, combined with clinicopathological factors, are associated with lymph node metastasis (LNM) in endometrial cancer (EC).

**Methods:**

This prospective study enrolled 73 patients with histologically confirmed EC and known nodal status. All underwent preoperative 3.0T MRI including diffusion-weighted imaging with 16 b-values. Thirteen parametric maps were derived from five non-Gaussian models: intravoxel incoherent motion (IVIM), diffusion kurtosis imaging (DKI), stretched exponential model (SEM), fractional-order calculus (FROC), and continuous time random walk (CTRW). Clinicopathological data were collected, including demographics, comorbidities, serum markers, pathological type, myometrial invasion, cervical involvement, and MRI-based local stage. Clinical, radiomics, and combined logistic regression models were built. Performance was evaluated using AUC, calibration curves, decision curve analysis, and bootstrap analysis of intercept variability.

**Results:**

LNM was confirmed in 21 of 73 patients. The clinical model achieved an AUC of 0.837, the radiomics model achieved an AUC of 0.856, and the combined model achieved an AUC of 0.924, with 90.5% sensitivity and 82.7% specificity. DeLong tests revealed no significant AUC differences among models (*P* = 0.090–0.140). The combined model had the lowest Brier score (0.092) and provided higher net benefit across threshold probabilities of 0.1–0.3.

**Conclusion:**

Non-Gaussian diffusion histogram parameters, particularly DKI_K_energy, demonstrate potential for predicting LNM in EC. A combined model incorporating DKI_K_energy with age and pathological type shows promise as a potential biomarker for risk stratification.

## 1 Introduction

Endometrial cancer (EC) is the sixth most common malignancy in women worldwide, with a steadily rising incidence (1–3). Endometrioid adenocarcinoma (EAC) comprises 80%–90% of EC cases and generally confers a favorable prognosis (4). Non-endometrioid carcinoma (NEAC), despite accounting for only approximately 10% of cases, drives >50% of recurrences and deaths (5). Lymph node metastasis (LNM) is one of the strongest prognostic factors in EC and directly influences decisions regarding adjuvant radiotherapy, chemotherapy, or combined treatment (6, 7). According to the International Federation of Gynecology and Obstetrics (FIGO) staging system, standard surgical staging includes hysterectomy, bilateral salpingo-oophorectomy, and systematic pelvic and para-aortic lymphadenectomy (8). However, systematic lymphadenectomy carries well-recognized risks of complications, including vascular injury, lymphocele, and lower-limb lymphedema (9), which may substantially impair quality of life (10–12). To reduce surgical morbidity, sentinel lymph node (SLN) biopsy has been increasingly adopted in place of systematic lymphadenectomy for patients with early-stage and low-risk EC. Nevertheless, nodal status uncertainty remains a concern in some patients. The false-negative rate of SLN biopsy alone is about 3% (13). For elderly patients, those with major comorbidities, or those at low preoperative risk, systematic lymphadenectomy may do more harm than good and is therefore unsuitable. Accurate evaluation of lymph node involvement remains a clinical challenge in patients with confirmed EC. In this context, MRI has become the standard for preoperative EC evaluation due to its excellent soft-tissue contrast.

MRI is the preferred imaging modality for preoperative staging of EC (14). However, conventional MRI relies primarily on size-based criteria for lymph node assessment, most commonly a short-axis diameter > 10 mm, which cannot reliably distinguish metastatic from inflammatory nodes (15). Conventional diffusion-weighted imaging (DWI) reflects the average water diffusion behavior within tissue via the apparent diffusion coefficient (ADC). Yet in highly heterogeneous tumors such as EC, water diffusion markedly departs from Gaussian behavior, and the mono-exponential ADC model is unable to account for these non-Gaussian characteristics, limiting its ability to accurately reflect microstructural complexity (16). Non-Gaussian diffusion models can characterize complex diffusion processes and may provide complementary information on tissue microstructure.

In recent years, several non-Gaussian diffusion models have been developed to more accurately characterize the complexity of water molecule motion in biological tissues. From a single multi-b-value DWI acquisition, five such models can be derived simultaneously: intravoxel incoherent motion (IVIM) (17), diffusion kurtosis imaging (DKI) (18), continuous time random walk (CTRW) (19), fractional-order calculus (FROC) (20), and stretched exponential model (SEM) (21). These models provide quantitative parameters reflecting microvascular perfusion, diffusion restriction, and spatial heterogeneity. Furthermore, whole-tumor histogram analysis captures the distributional characteristics of voxel values across the entire lesion, offering a comprehensive assessment of tumor heterogeneity while reducing the sampling bias inherent in single-slice region-of-interest placement (22–25).

Although Yamada et al. first associated DKI parameters with LNM, myometrial invasion depth, and grade in EC, their single-slice ROI analysis lacked comparison with other non-Gaussian models (26). More recently, Bi et al. compared six diffusion models for EC vs. benign lesion discrimination, identifying MK, D, and αCTRW as independent predictors (27). Critically, their study addressed differential diagnosis rather than LNM prediction in pathologically confirmed EC. However, the value of these advanced diffusion techniques in assessing LNM in EC, particularly using whole-tumor histogram analysis, remains to be fully characterized.

For further exploration, this study systematically evaluated five non-Gaussian diffusion models, namely IVIM, DKI, SEM, FROC, and CTRW, and integrated the resulting histogram features with routine clinicopathological indicators. The primary objective was to investigate the potential of these integrated parameters in assessing whether EC patients have LNM, offering a new perspective for clinical staging and therapeutic decision-making.

## 2 Materials and methods

### 2.1 Study participants

This study was approved by the Ethics Committee of The First Hospital of Henan Medical University (approval No. EC-025-131) and complied with the Declaration of Helsinki. Written informed consent was obtained from all participants. Consecutive patients with suspected endometrial cancer (*n* = 96) who completed conventional MRI and multi-b-value (0–3,600 s/mm^2^) DWI within 2 weeks before surgery were enrolled between September 2024 and December 2025. Inclusion and exclusion criteria are presented in Figure 1. A total of 73 cases of LNM positive (*n* = 21) and LNM negative (*n* = 52) were included in this study.

**Figure 1:**
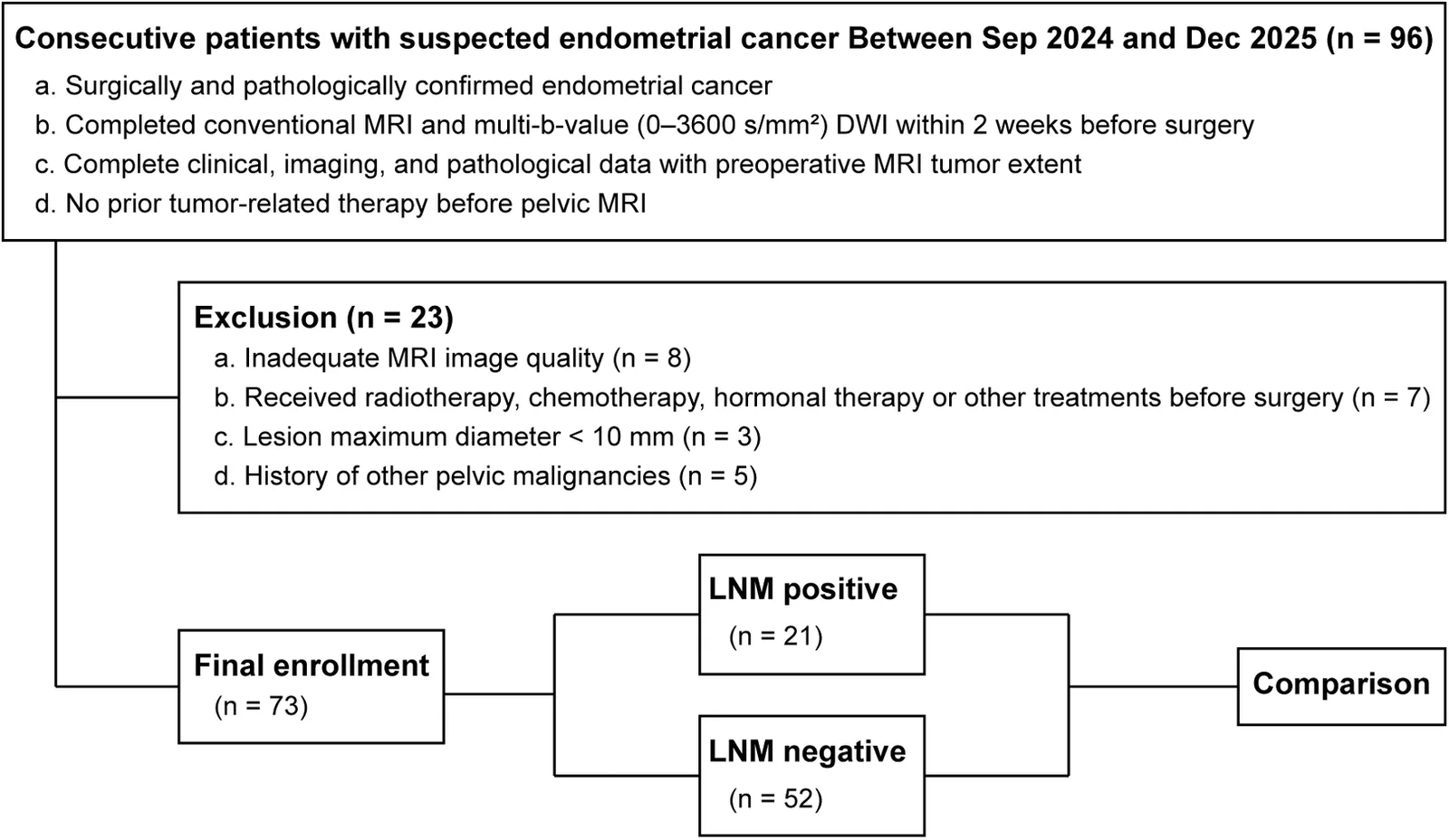
Flowchart of participants’ selection process.

### 2.2 Clinical and pathological data

Baseline clinical data (age, BMI, menopausal status, hypertension, and diabetes) were extracted from electronic medical records. Serum levels of CA125 and CA199 were determined within one week prior to surgery, with normal reference ranges of 0–35 U/mL and 0–27 U/mL, respectively. Values above the aforementioned thresholds were defined as positive. Preoperative tumor extension was assessed on MRI and classified as MRI-based local stage, determined solely by the anatomical extent of the primary tumor on MRI and specifically excluding nodal status. MRI-based local stage was assessed by two independent radiologists with 15 and 10 years of experience, who were blinded to clinical and pathological data. MRI-based local stage I was defined as tumor confined to the uterine corpus without cervical stromal invasion or extrauterine extension. MRI-based local stage II/III was defined as cervical stromal invasion or extension beyond the uterus, including uterine serosa, adnexa, vagina, parametria, or pelvic peritoneum. The LNM reference standard was postoperative pathological examination. All patients underwent systematic pelvic and para-aortic lymphadenectomy. Para-aortic lymph node dissection was routinely included. Isolated tumor cells and micrometastases were considered as positive. Pathologists were blinded to preoperative MRI findings. Pathological type (EAC/NEAC), depth of myometrial invasion (<1/2 or ≥1/2), and cervical involvement were determined from postoperative hysterectomy specimens and corresponding pathological reports in patients who underwent standard surgical staging.

### 2.3 MRI acquisition

Pelvic MRI was performed using a 3.0T scanner (Discovery MR750, GE Healthcare, Milwaukee, Wisconsin). The protocol included sagittal T2 weighted imaging (T2WI), axial oblique T2WI (perpendicular to the long axis of the uterus), conventional axial DWI (*b* = 0, 800 s/mm^2^) and multi b value DWI. Multi b value DWI used a spin-echo-echo-planar imaging (SE-EPI) sequence with the following parameters: TR = 6,420 ms, TE = 67 ms, FOV = 420 × 420 mm^2^, matrix = 384 × 128, slice thickness = 4.0 mm. B-values were: 0, 50, 80, 100, 150, 200, 300, 500, 800, 1,000, 1,300, 1,700, 1,800, 2,400, 3,000 and 3,600 s/mm^2^.

### 2.4 Image processing

A total of 13 parametric maps corresponding to five non-Gaussian diffusion models were simultaneously generated from the multi-b-value DWI data using an in-house Python-based software. The output parameters comprised: (1) IVIM (f, D, D*); (2) DKI (K, D); (3) SEM (α, DDC); (4) FROC (β, μ, D); (5) CTRW (α, β, D). The mathematical formulations of these models are summarized below (28–30). The fitting procedure is described in full detail in the Supplementary Methods.

(1) IVIM model:
  $$S=S_{0}[\;f\;\exp(-bD^{*})+(1-f)\exp(-bD)]$$

The parameter *f* reflects the perfusion fraction contributed by microvascular flow, D* describes the pseudo-diffusion behavior of blood within capillaries, and D quantifies the pure diffusivity of water molecules following the removal of perfusion-related signal components.

(2) DKI model:
  $$S=S_{0}\exp\left[-bD+\frac{1}{6}K{\left(bD\right)}^{2}\right]$$

The parameter K quantifies kurtosis, which reflects the degree of microstructural complexity in tissues, whereas D denotes the diffusivity adjusted to account for non-Gaussian water motion.

(3) SEM model:
  $$S=S_{0}\exp[{\left(-b\cdot DDC\right)}^{\alpha }]$$

The parameter α represents the stretching exponent, which quantifies the extent of departure from Gaussian diffusion, while DDC (distributed diffusion coefficient) characterizes the overall rate of non-Gaussian diffusion.

(4) FROC model:
  $$S=S_{0}\;\exp\left[-D\mu^{2(\beta -1)}{\left(\gamma G_{d}\delta \right)}^{2\beta }\left(\Delta -\frac{2\beta -1}{2\beta +1}\delta \right)\right]$$

The parameter β denotes the fractional order derivative in space, which reflects spatial heterogeneity of diffusion, while μ is an auxiliary spatial parameter introduced to maintain the unit consistency of D (mm^2^/s).

(5) CTRW model:
  $$S=S_{0}E_{\alpha }[-(bD^{\beta })]$$

The parameter E_α_ represents the Mittag-Leffler function, while α and β describe the waiting time and step length distributions of water molecule movement, respectively, and D denotes the anomalous diffusion coefficient, which reflects spatiotemporal heterogeneity.

### 2.5 Tumor segmentation and histogram analysis

Two radiologists (15 and 10 years of pelvic MRI experience, blinded to clinical/pathological data) independently delineated the volume of interest (VOI) slice-by-slice on DWI (*b* = 800) using 3D Slicer, with axial T2WI as anatomical reference. Parametric maps from five non-Gaussian diffusion models were rigidly co-registered to DWI space via mutual information, and each resulting VOI was copied to all maps. Disagreements were resolved by consensus, with the senior radiologist adjudicating when needed. Final histogram features per patient were averages of extractions by both radiologists. No histogram features were derived from DWI or T2WI. Using Py-Radiomics (v3.0.1) in Python 3.9, 16 features per parametric map were extracted: mean, median, maximum, minimum, range, mean absolute deviation, robust mean absolute deviation, root mean squared, skewness, kurtosis, variance, energy, total energy, interquartile range, and 10th/90th percentiles, yielding 208 features per patient. Representative EC cases without and with LNM are presented in Figures 2, 3, respectively.

**Figure 2:**
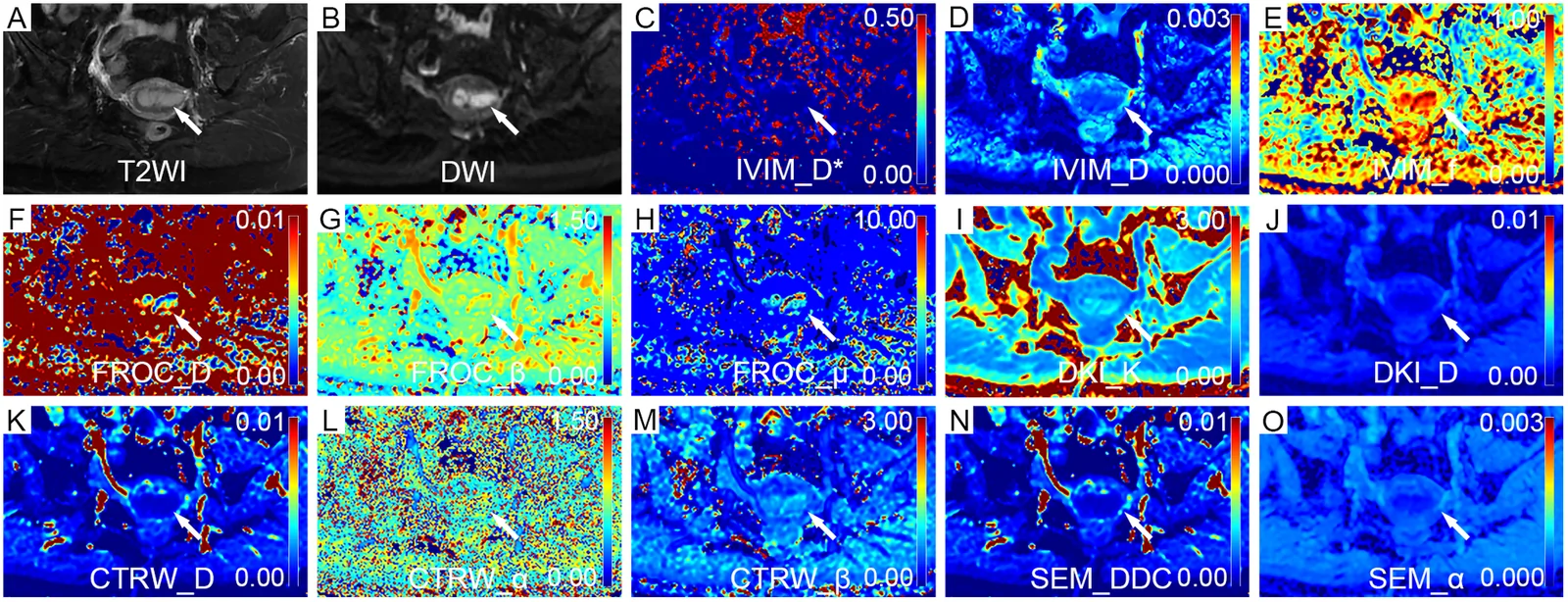
Conventional sequences and parametric maps derived from five diffusion-weighted MRI models in a 55-year-old female with EC and negative LNM. **(A)** T2-weighted image (T2WI); **(B)** diffusion-weighted image (DWI); **(C)** IVIM_D* map; **(D)** IVIM_D map; **(E)** IVIM_f map; **(F)** FROC_D map; **(G)** FROC_β map; **(H)** FROC_µ map; **(I)** DKI_K map; **(J)** DKI_D map; **(K)** CTRW_D map; **(L)** CTRW_α map; **(M)** CTRW_β map; **(N)** SEM_DDC map; **(O)** SEM_α map. All parametric maps are presented in pseudo-color.

**Figure 3:**
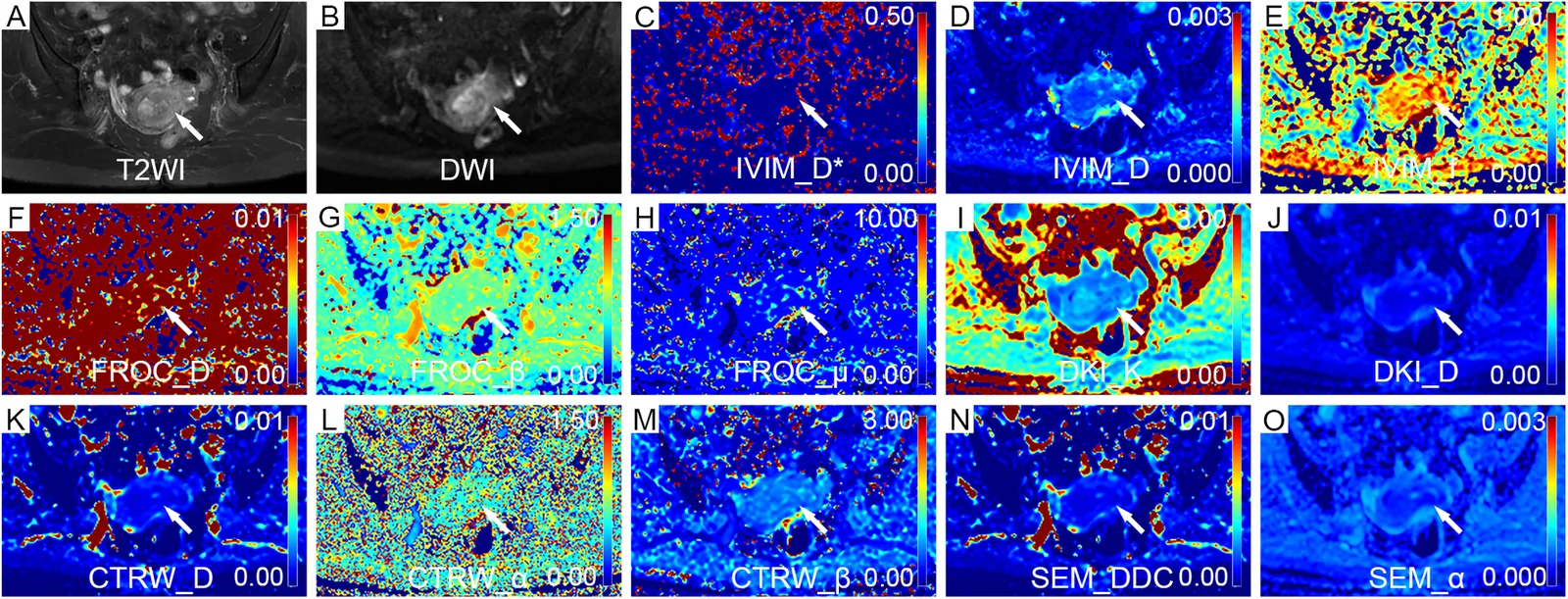
Conventional sequences and parametric maps derived from five diffusion-weighted MRI models in a 60-year-old female with EC and positive LNM. **(A)** T2-weighted image (T2WI); **(B)** diffusion-weighted image (DWI); **(C)** IVIM_D* map; **(D)** IVIM_D map; **(E)** IVIM_f map; **(F)** FROC_D map; **(G)** FROC_β map; **(H)** FROC_µ map; **(I)** DKI_K map; **(J)** DKI_D map; **(K)** CTRW_D map; **(L)** CTRW_α map; **(M)** CTRW_β map; **(N)** SEM_DDC map; **(O)** SEM_α map. All parametric maps are presented in pseudo-color.

### 2.6 Statistical analysis

Statistical analyses used SPSS 27.0 and MedCalc 15.6.1. Inter-observer agreement for quantitative variables was evaluated using the intraclass correlation coefficient (ICC), categorized as poor (0.00–0.50), moderate (0.51–0.75), good (0.76–0.90), and excellent (0.91–1.00) (31). And the categorical MRI-local tumor extent was assessed using Cohen's Kappa. Normally distributed continuous variables expressed as mean ± SD and compared by independent *t*-test; non-normal ones as median (Q1, Q3) and compared by Mann-Whitney *U* test. Categorical data reported as counts (%) and compared by *χ*^2^ or Fisher's exact test as appropriate.

Feature selection from 208 histogram features involved: univariate Mann-Whitney *U* test (retaining *P* < 0.01), followed by LASSO regression with 10-fold cross-validation, with optimal λ determined by minimum deviance. The selected features were evaluated using Spearman correlation and collinearity diagnostics, specifically tolerance (TOL) and variance inflation factor (VIF), to exclude those with high multicollinearity.

Three logistic regression models (clinical, radiomics, combined) were built. Discriminative ability assessed by receiver operating characteristic (ROC) curves with area under the curve (AUC) values compared by DeLong test. Additional evaluations included calibration curves, Brier score, decision curve analysis, and bootstrap analysis of intercept estimates. A two-sided *P* < 0.05 was considered statistically significant.

## 3 Results

### 3.1 Clinical baseline characteristics

Among the 73 patients (median age, 59 years, range, 33–90 years), 21 were LNM-positive and 52 were LNM-negative. Compared with the LNM-negative group, LNM-positive patients had a more aggressive clinicopathological profile, with older age and higher proportions of NEAC, cervical involvement, deep myometrial invasion (>1/2), preoperative MRI-based local stage II/III disease, and CA125/CA199 positivity (all *P* < 0.05). No significant between-group differences were observed in BMI, menopausal status, hypertension, or diabetes mellitus (Table 1).

**Table 1:** Clinical and pathological characteristics of patients stratified by LNM status.

Parameters	LNM (−) (*n* = 52)	LNM (+) (*n* = 21)	*P*
Age, year	57.5 (54.0, 65.0)	66.0 (57.0, 72.5)	**0**.**006**
BMI, kg/m^2^	26.7 ± 3.6	25.8 ± 3.2	0.301
Pathological type, *n* (%)			**0**.**001**
EAC	48 (92.3)	11 (52.4)	
NEAC	4 (7.7)	10 (47.6)	
Menopause, *n* (%)			0.679
Yes	47 (90.4)	19 (90.5)	
No	5 (9.6)	2 (9.5)	
Hypertension, *n* (%)			0.138
Yes	22 (42.3)	5 (23.8)	
No	30 (57.7)	16 (76.2)	
Diabetes mellitus, *n* (%)			0.402
Yes	11 (21.2)	2 (9.5)	
No	41 (78.8)	19 (90.5)	
Cervical involvement, *n* (%)			**0**.**037**
Yes	10 (19.2)	9 (42.9)	
No	42 (80.8)	12 (57.1)	
Depth of myometrial, *n* (%)			**0**.**005**
<1/2	36 (69.2)	7 (33.3)	
≥1/2	16 (30.8)	14 (66.7)	
MRI-based local stage, *n* (%)			**0**.**003**
Stage I	27 (51.9)	3 (14.3)	
Stage II/III	25 (48.1)	18 (85.7)	
CA125 status, *n* (%)			**0**.**015**
+	14 (26.9)	12 (57.1)	
−	38 (73.1)	9 (42.9)	
CA199 status, *n* (%)			**0**.**024**
+	11 (21.2)	10 (47.6)	
−	41 (78.8)	11 (52.4)	

MRI-based local stage refers to the preoperative MRI-defined extent of the primary tumor, excluding LNM status.

BMI, body mass index; EAC, endometrioid adenocarcinoma; NEAC, non-endometrioid endometrial carcinoma; CA125, cancer antigen 125; CA199, carbohydrate antigen 199; LNM, lymph node metastasis.

*P* values < 0.05 are shown in bold.

### 3.2 Feature selection

The ICCs for the diffusion parameters in the study ranged from moderate to excellent agreement between the two observers (ICC: 0.706–0.993) (Supplementary Table S1). And Inter-reader agreement for MRI-local tumor extent was *κ* = 0.890 (Supplementary Table S2). From the 208 histogram features, 29 with *P* < 0.01 were retained and subjected to LASSO regression, which selected three radiomics features: DKI_K_energy, FROC_μ_10th percentile, and DKI_K_total energy. Compared with the LNM-negative group, the LNM positive group showed higher DKI_K_energy [1,800.544 (471.717, 2,953.585) *vs.* 415.358 (183.693, 717.209)], higher FROC_μ_10th percentile [1.000 (1.000, 1.220) *vs.* 1.000 (1.000, 1.000)], and higher DKI_K_total energy [12,660.167 (3,316.749, 20,767.452) *vs.* 2,920.202 (1,291.583, 5,042.877)] in Figure 4 and Supplementary Figure S1.

**Figure 4:**
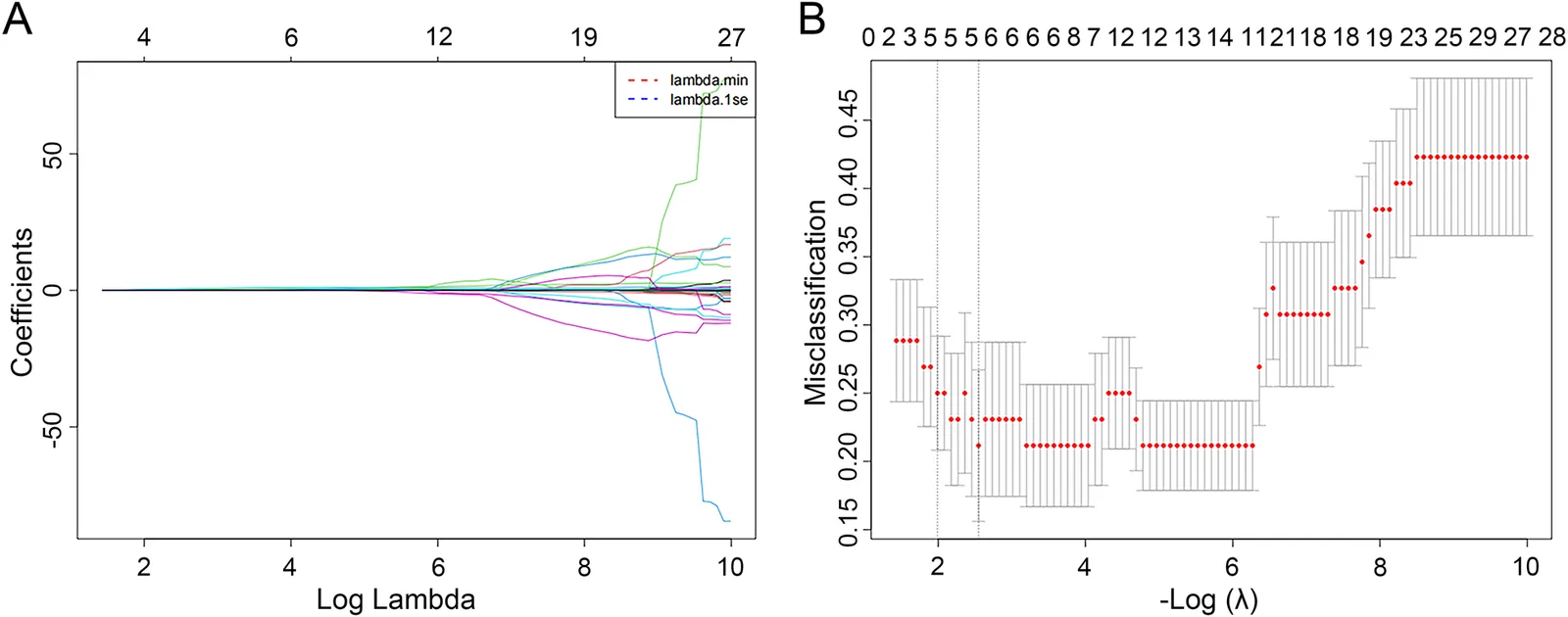
LASSO regression analysis for selecting histogram features associated with LNM. **(A)** Cross-validation curve for selecting the optimal penalty parameter λ. **(B)** LASSO coefficient profiles of the candidate histogram features.

### 3.3 Spearman correlation analysis and collinearity diagnostics

Spearman correlation analysis revealed that LNM showed moderate or stronger correlations (|r| ≥ 0.30, all *P* < 0.05) with depth of myometrial invasion (*r* = −0.459), DKI_K_energy (*r* = 0.478), DKI_K_total energy (*r* = 0.478), FROC_μ_10th percentile (*r* = 0.376), MRI-based local stage (*r* = 0.346), cervical involvement (*r* = 0.330), and age (*r* = 0.325); and weakly correlated with CA125 (*r* = 0.286), CA199 (*r* = 0.265), and pathological type (*r* = 0.244) (0.20 ≤ |r| <0.30, all *P* < 0.05). A significant positive correlation was observed between CA125 and CA199 (*r* = 0.602, *P* < 0.05). DKI_K_energy and DKI_K_total energy exhibited perfect positive correlation (*r* = 1.000, *P* < 0.05). No notable correlations were found among the remaining variables (|r| < 0.20).

Collinearity diagnostics confirmed severe collinearity between DKI_K_energy (VIF = 136.639, TOL = 0.007) and DKI_K_total energy (VIF = 135.053, TOL = 0.007). Both VIF values far exceeded 10 and TOL values were below 0.1. Because total energy accumulates over the entire three-dimensional volume, DKI_K_total energy scales proportionally to DKI_K_energy. DKI_K_energy and DKI_K_total energy were perfectly correlated (*r* = 1.000, VIF > 135) and showed identical univariate performance (AUC = 0.805). Therefore, DKI_K_total energy was excluded to avoid redundancy, and DKI_K_energy was retained. Other Parameters showed acceptable collinearity. Despite its significance in univariate analysis, cervical involvement yielded AUC: 0.618 (*P* = 0.116) and was consequently excluded from further model development in Table 2 and Figure 5.

**Table 2:** Multicollinearity diagnostics of candidate clinical and histogram features.

Parameters	TOL	VIF
Age	0.798	1.253
Pathological type	0.579	1.728
Depth of myometrial	0.660	1.515
Cervical involvement	0.783	1.277
MRI-based local stage	0.797	1.254
CA125 status	0.522	1.915
CA199 status	0.497	2.012
DKI_K_energy	0.007	136.639
FROC_μ_10th percentile*	0.851	1.175
DKI_K_total energy	0.007	135.053

MRI-based local stage refers to the preoperative MRI-defined extent of the primary tumor, excluding LNM status. CA125, cancer antigen 125; CA199, carbohydrate antigen 199.

TOL, tolerance; VIF, variance inflation factor; DKI, diffusion kurtosis imaging; FROC, fractional-order calculus.

* ×10⁻^3^ mm.

**Figure 5:**
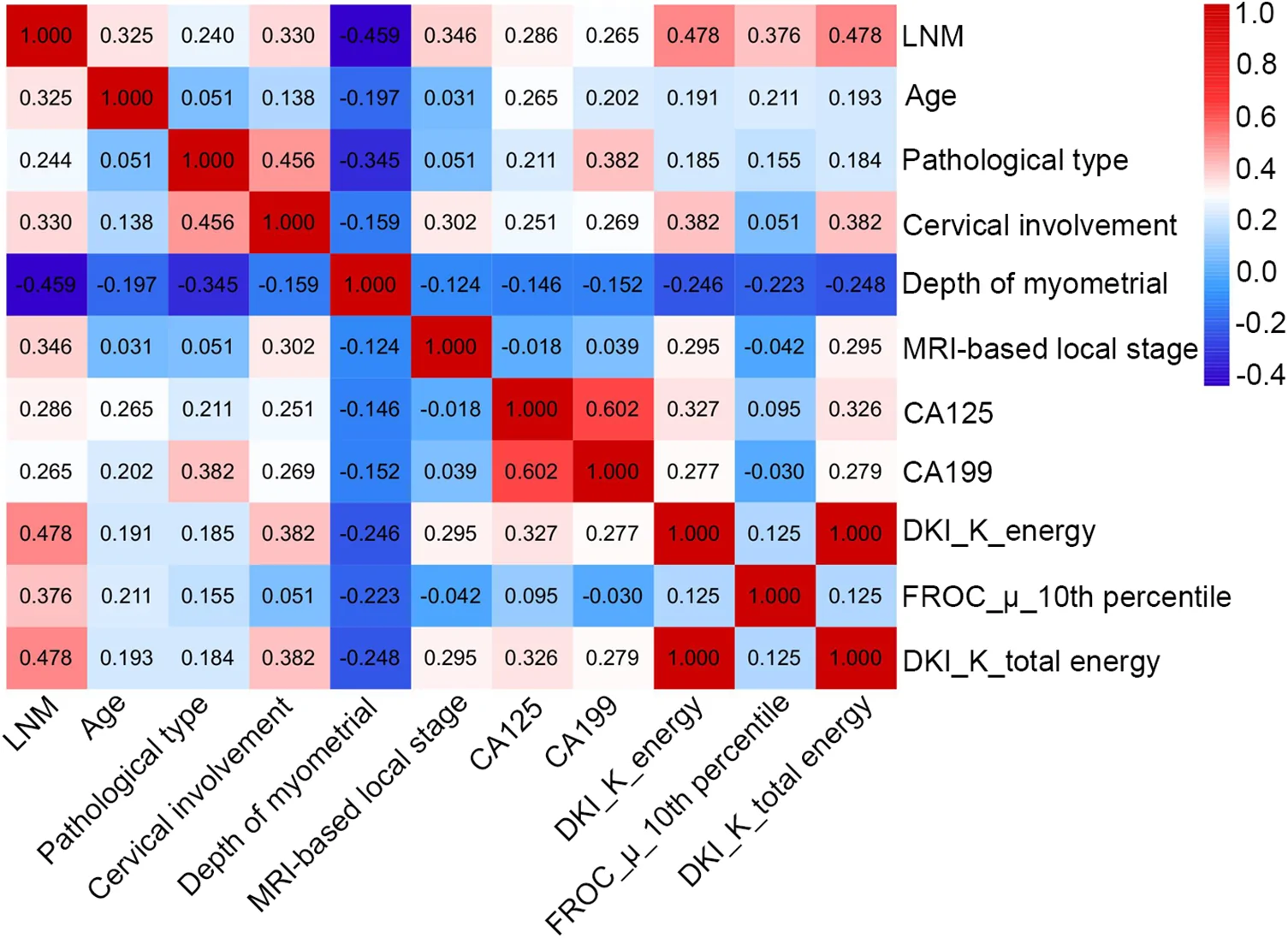
Spearman correlation heatmap of candidate clinical, pathological, and histogram features. Color intensity represents the Spearman correlation coefficient. Correlation strength was defined as |r| < 0.20, negligible; 0.20 ≤ |r| < 0.30, weak; and |r| ≥ 0.30, moderate or stronger. MRI-based local stage refers to the preoperative MRI-defined extent of the primary tumor, excluding LNM status. CA125, cancer antigen 125; CA199, carbohydrate antigen 199; DKI, diffusion kurtosis imaging; FROC, fractional-order calculus.

### 3.4 Univariate performance for LNM assessment

Univariate ROC analysis was performed to evaluate the diagnostic performance of individual variables for identifying LNM. Among the histogram features, DKI_K_energy showed the highest discriminative ability (AUC = 0.805, 95% CI: 0.690–0.920, *P* < 0.001; sensitivity 61.9%, specificity 92.3%), followed by FROC_μ_10th percentile (AUC = 0.655, 95% CI: 0.506–0.803, *P* = 0.039). Among the clinical, pathological, and MRI-based staging variables, age, pathological type, depth of myometrial invasion, preoperative MRI-based local stage, and CA125 showed significant discriminative performance for identifying LNM, with AUCs of 0.707, 0.700, 0.679, 0.688, and 0.651, respectively (all *P* < 0.05). The remaining variables, including cervical stromal involvement and CA199, did not reach statistical significance in univariate ROC analysis in Table 3 and Figure 6.

**Table 3:** Univariate ROC analysis of candidate variables for LNM assessment.

Parameters	Cut-off value	Youden index	Sensitivity	Specificity	AUC	95% CI	*P*
Age	65.5	0.332	0.524	0.808	0.707	0.571–0.843	**0**.**006**
Pathological type	0.5	0.399	0.476	0.923	0.700	0.553–0.846	**0**.**008**
Depth of myometrial	0.5	0.359	0.667	0.692	0.679	0.541–0.818	**0**.**017**
Cervical involvement	0.5	0.237	0.429	0.808	0.618	0.469–0.767	0.116
MRI-based local stage	0.5	0.376	0.857	0.519	0.688	0.560–0.816	**0**.**012**
CA125 status	0.5	0.302	0.571	0.731	0.651	0.508–0.795	**0**.**044**
CA199 status	0.5	0.264	0.476	0.788	0.632	0.485–0.780	0.078
DKI_K_energy	1,186.0	0.542	0.619	0.923	0.805	0.690–0.920	**<0**.**001**
FROC_μ_10th percentile*	1.1	0.295	0.333	0.962	0.655	0.506–0.803	**0**.**039**
DKI_K_total energy	8,339.0	0.542	0.619	0.923	0.805	0.690–0.920	**<0**.**001**

For all variables, sensitivity and specificity were calculated with LNM-positive as the event of interest. Pathological type was coded as NEAC = 1 and EAC = 0. MRI-based local stage was coded as stage II/III = 1 and stage *I* = 0. CA125 and CA199 were coded as positive = 1 and negative = 0.

ROC, receiver operating characteristic; AUC, area under the curve; CI, confidence interval; LNM, lymph node metastasis. MRI-based local stage referred to the preoperative MRI-defined extent of the primary tumor, excluding LNM status. CA125, cancer antigen 125; CA199, carbohydrate antigen 199; DKI, diffusion kurtosis imaging; FROC, fractional-order calculus.

* ×10⁻^3^ mm.

*P* values < 0.05 are shown in bold.

**Figure 6:**
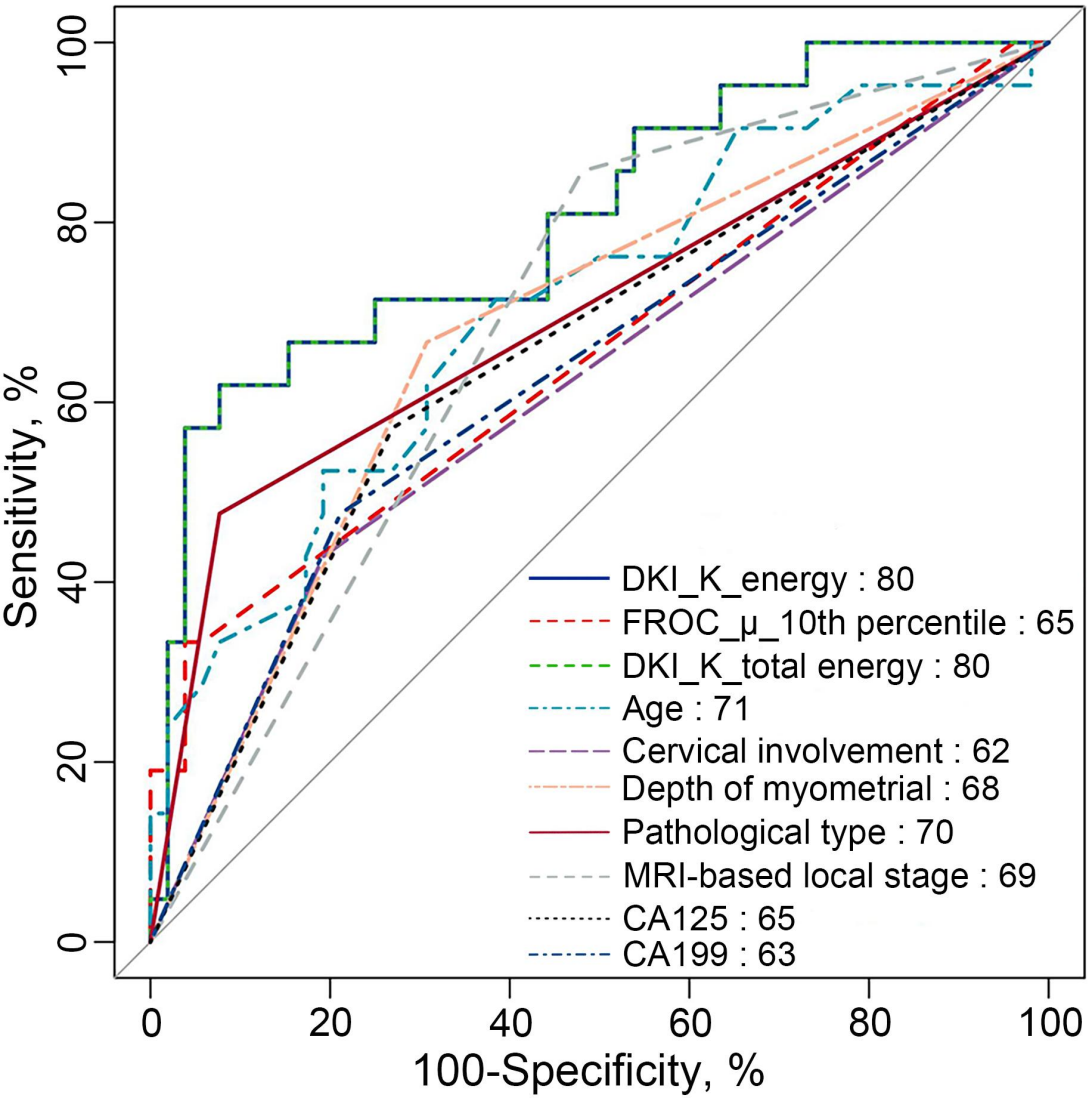
ROC curves of individual clinical, pathological, MRI-local tumor-extent, and histogram variables for LNM assessment. AUC, area under the curve; ROC, receiver operating characteristic; LNM, lymph node metastasis; CA125, cancer antigen 125; CA199, carbohydrate antigen 199; DKI, diffusion kurtosis imaging; FROC, fractional-order calculus. MRI-based local stage refers to the preoperative MRI-defined extent of the primary tumor, excluding LNM status.

### 3.5 Construction of LNM assessment models

Based on Spearman correlation analysis and collinearity diagnostics, three LNM assessment models were established: Clinical model (M-clinical): age + pathological type + preoperative MRI-based local stage; Radiomics model (M-radiomics): DKI_K_energy + FROC_μ_10th percentile; Combined model (M-combined): DKI_K_energy + age + pathological type. No notable collinearity was found among variables in any model (Supplementary Table S3). M-clinical included routine clinical factors, pathological type, and MRI-local tumor-extent information. The regression coefficients, odds ratios, 95% CIs, and *P* values for all variables across the three models are presented in Supplementary Table S5, with the full regression equations provided in the Supplementary Methods.

### 3.6 Diagnostic performance of the LNM assessment models

Among the three models, the combined model achieved the highest AUC (0.924, 95% CI: 0.857–0.991; sensitivity 90.5%, specificity 82.7%), followed by radiomics (AUC: 0.856, 95% CI: 0.756–0.957) and clinical (AUC: 0.837, 95% CI: 0.735–0.939) models. The combined model had the highest sensitivity, while the radiomics and clinical models both had specificity 0.923. DeLong test showed no significant AUC differences between combined and radiomics (*P* = 0.090) or clinical (*P* = 0.140). Therefore, the combined model was not significantly superior to individual models in Tables 4, 5.

**Table 4:** Apparent performance of the clinical, radiomics, and combined models for LNM assessment.

Parameters	Cut-off value	Youden index	Sensitivity	Specificity	AUC	95% CI	*P*
M-clinical	0.394	0.542	0.619	0.923	0.837	0.735–0.939	<0.05
M-radiomics	0.379	0.637	0.714	0.923	0.856	0.756–0.957	<0.05
M-combined	0.193	0.732	0.905	0.827	0.924	0.857–0.991	<0.05

M-clinical, clinical model; M-radiomics, radiomics model; M-combined, combined model. AUC, area under the curve; CI, confidence interval.

**Table 5:** Pairwise comparison of model AUCs using the DeLong test.

Parameters	Difference in AUC	*Z*	95% CI	*P*
M-combined *vs.* M-radiomics	0.068	1.693	−0.011–0.146	0.090
M-combined *vs.* M-clinical	0.087	1.476	−0.029–0.203	0.140
M-radiomics *vs.* M-clinical	0.019	0.234	−0.142–0.180	0.815

M-clinical, clinical model; M-radiomics, radiomics model; M-combined, combined model. AUC, area under the curve; CI, confidence interval.

### 3.7 Calibration, decision curve analysis, and stability assessment

Despite non-significant AUC differences, the combined model outperformed the others in calibration and net benefit (Figure 7), with a Brier score of 0.092, lower than clinical (0.129) and radiomics (0.120), indicating better probability–outcome agreement. Decision curve analysis showed that across threshold probabilities of 0.1–0.3, the combined model consistently had higher net benefit than the other two models and the treat all or treat none strategies. Bootstrap analysis was used only to estimate the variability of the intercept in the three models and should not be interpreted as validation of model performance. (Supplementary Table S4).

**Figure 7:**
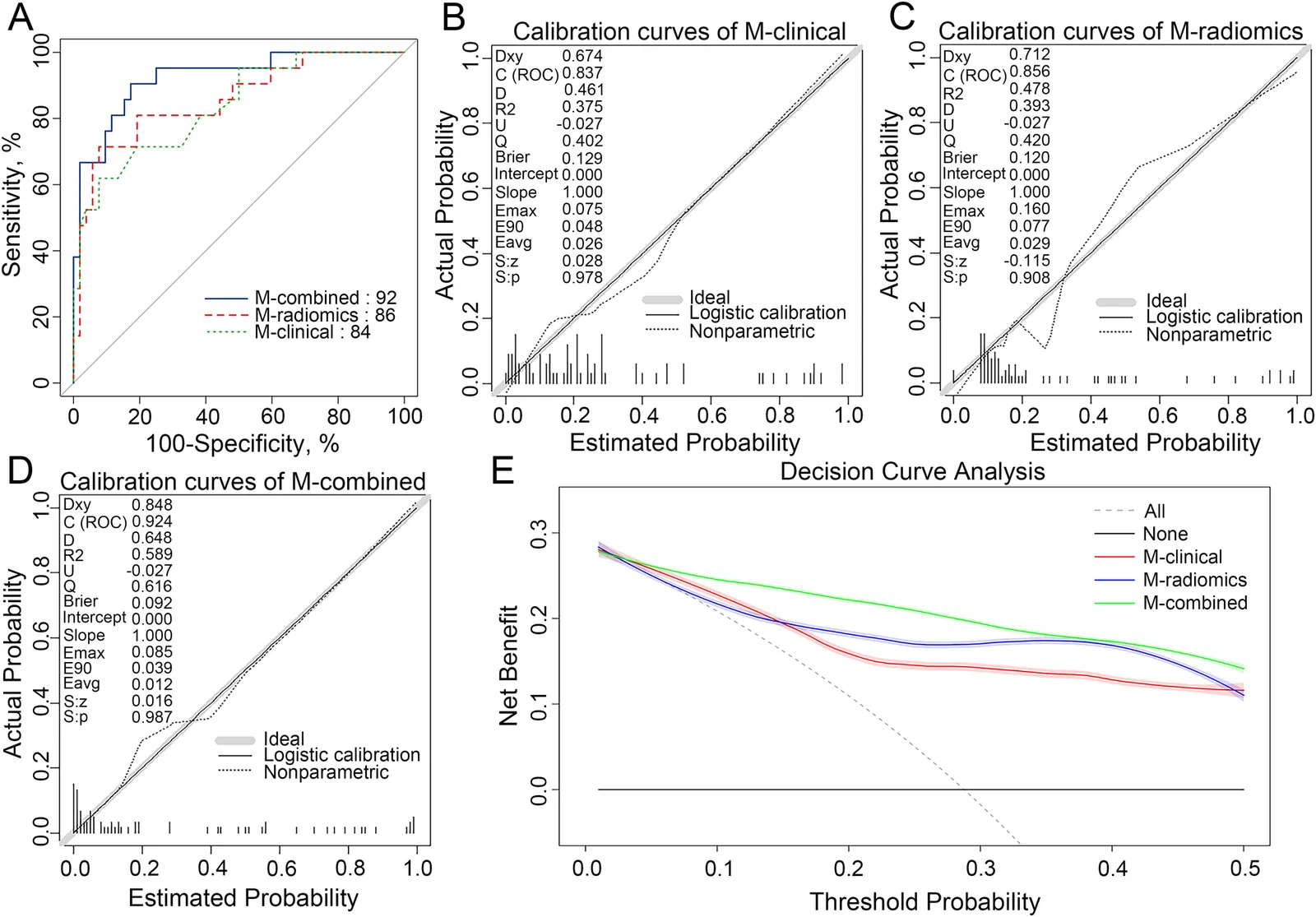
ROC curves, calibration curves, and decision curve analysis of the clinical, radiomics, and combined models for assessing LNM. **(A)** ROC curves: area under the curve (AUC) values for M-clinical, M-radiomics, and M-combined are 0.84, 0.86, and 0.92, respectively. **(B)** Calibration curve of M-clinical. **(C)** Calibration curve of M-radiomics. **(D)** Calibration curve of M-combined. **(E)** Decision curve analysis showing the net benefit of the three models across different threshold probabilities, with shaded areas representing 95% confidence intervals. M-clinical, clinical model; M-radiomics, radiomics model; M-combined, combined model; LNM, lymph node metastasis; ROC, receiver operating characteristic; AUC, area under the curve.

## 4 Discussion

Lymph node status is now regarded as a critical prognostic factor for EC patients and is closely related to treatment decisions and clinical outcomes. Several clinicopathological factors were identified as being associated with LNM. Previous studies have linked age and menopausal status to EC risk, particularly in postmenopausal women and women older than 50 years (32, 33). In this study, the optimal age cut-off was 65.5 years, whereas menopausal status was not significant, suggesting that age may better reflect the age-related increase in metastatic risk. However, its modest univariate AUC of 0.707 indicates that age should be used only as an auxiliary factor. NEAC, an aggressive histological subtype, was also retained in the final model, consistent with previous reports (34). In addition, deep myometrial invasion, MRI-based local stage II/III disease, and elevated tumor markers were more common in LNM-positive patients. These findings suggest that aggressive histology, local tumor extension, and increased tumor burden may indicate higher lymphatic dissemination potential (33).

Beyond these clinical factors, radiomic features derived from the five non-Gaussian diffusion models were also analyzed for their associations with LNM. The FROC_μ parameter reflects spatial heterogeneity of water diffusion; μ is inversely related to mean diffusion length, and active proliferation shortens the diffusion distance, raising μ in malignancies (35). Histogram percentile meanings differ: low-percentile metrics capture diffusion-restricted or high-cellular subregions, while high-percentile ones may reflect cysts, necrosis, or secretions (36). We found elevated FROC_μ_10th in LNM-positive tumors, suggesting that μ values may be higher even at low percentiles. Since higher μ signifies greater restriction, this suggests LNM-positive tumors contain more prominent high-cellular, disorganized regions with restricted diffusion, consistent with low-percentile parameters reflecting high density and aggressiveness (37–39).

Unlike the FROC_μ parameter, the DKI_K parameter, representing mean diffusion kurtosis, positively correlates with microstructural complexity and heterogeneity (40). Prior studies show K and D assess invasion depth, LNM, and grade, consistent with our results (26, 41). At whole-tumor histogram level, DKI_K_energy was higher in LNM-positive patients. Energy quantifies gray-level uniformity, with lower energy indicating higher heterogeneity (42). Tumor heterogeneity links to malignancy and invasiveness. Yang et al. found higher T2-energy in rectal cancer without LNM (43). However, higher DKI_K_energy in LNM-positive EC likely reflects dense, uniform high-cellularity rather than necrosis or cysts, analogous to malignant testicular tumors showing higher energy than benign ones (22). Thus, the biological meaning of energy depends on tumor type, sequence, and diffusion model. Collectively, these findings support whole-tumor histogram analysis for reducing ROI sampling bias and comprehensively capturing microstructural heterogeneity.

The combined model integrated DKI_K_energy, age, and pathological type. The DeLong test showed no significant improvement in AUC compared with the other two models. Nevertheless, the combined model showed acceptable calibration and net benefit. Of note, FROC_μ_10th percentile was not included in the final model. It lost significance after adjustment for clinical covariates. Its addition also did not improve model parsimony. This pattern has been seen in earlier radiomics studies. In these studies, combined models improved net benefit but did not always show a significant AUC gain. Tao et al. developed a multiparametric MRI-based nomogram for EC LNM prediction and found that the combined model improved net benefit, but its AUC advantage was not always significant (44). Liu et al. constructed a nomogram combining ultrasound radiomics and clinical features, and reported good clinical utility with only modest improvement over the clinical-only model (45). These findings suggest that AUC may not fully reflect the value of adding radiomic features. This is especially true when the clinical model already performs well. The better calibration and net benefit of our combined model likely come from the complementary value of DKI_K_energy and clinical factors. DKI_K_energy reflects the distribution characteristics of non-Gaussian water-diffusion signals within the tumor. Age and pathological type capture host and tumor characteristics. Their integration offers a more balanced risk assessment. Nevertheless, the added discriminative value needs confirmation in independent external cohorts.

Several limitations should be acknowledged. First, the study was not registered, no *a priori* sample-size calculation was performed, and it was a single-centre prospective study with a small and imbalanced sample. As no optimism-corrected internal validation was performed, the reported performance metrics should be regarded as apparent estimates. Hence, generalisability requires multicentre validation with larger cohorts. Moreover, we did not perform conventional ADC histogram analysis and therefore could not establish the superiority of non-Gaussian models over conventional diffusion, and future work should assess their incremental value. Second, the cohort was restricted to patients selected for nodal staging, resulting in a higher LNM prevalence than in unselected EC populations. This may limit the generalisability of the models to unselected preoperative populations. Third, motion correction and EPI distortion correction were not applied, which may affect the accuracy of diffusion parameter estimation, particularly at high b-values. In addition, manual tumor segmentation introduces subjective bias. Automated or AI-based segmentation could reduce variability and improve objectivity. Lastly, the calibration and net-benefit advantages of the combined model are exploratory findings. Independent external validation is required before clinical application.

In this study, we compared the performance of five non-Gaussian diffusion models for evaluating LNM status in EC patients. The results demonstrated that the combined model provided better diagnostic performance. The integration of DKI_K_energy with clinical factors offered multidimensional information on tumor biology and host characteristics, yielding valuable complementary insights. Therefore, the use of multi-model diffusion imaging combined with clinical indicators may provide better diagnostic support, enabling clinicians to obtain comprehensive tumor information and thereby facilitating more informed risk stratification.

## 5 Conclusion

This exploratory study demonstrates that whole-tumor histogram analysis of non-Gaussian diffusion MRI generates quantitative features associated with LNM in EC. Among the five models evaluated, DKI_K_energy showed the strongest association with LNM status. A combined model incorporating DKI_K_energy with age and pathological type achieved satisfactory calibration and net clinical benefit. These findings demonstrate the potential of multi-b-value non-Gaussian diffusion models combined with clinical indicators in predicting LNM in EC, which may help refine risk stratification and guide individualized treatment decisions.

## Data Availability

The original contributions presented in the study are included in the article/Supplementary Material, further inquiries can be directed to the corresponding author.
