# Supplementary material for: Whole-tumor histogram analysis of non-Gaussian diffusion MRI models combined with clinicopathological indicators for assessing lymph node metastasis in endometrial cancer
Source: Front Med (Lausanne). 2026 Oct 22;13:1959334. doi: 10.3389/fmed.2026.1959334 (PMC13736822; doi:10.3389/fmed.2026.1959334)
Supplement: Supplementary file 1 [file Supplementaryfile1.docx]

Supplementary Material

**1. Supplementary Methods**

**1.1 Diffusion modeling and fitting procedure**

The DWI data were preprocessed prior to fitting. No motion correction, EPI distortion correction, or noise-floor correction was applied in the current pipeline. A slice-wise 3×3 mean filter was applied before fitting to reduce high-b-value noise. All signals were normalized to the mean b0 signal, and b-values <20 s/mm² were treated as b0.

Fitting was performed using an in-house Python script. For DKI, log-domain linear least squares were used, with D constrained to 0–0.01 mm²/s and K constrained to 0–3. For IVIM, SEM, FROC, and CTRW, bounded nonlinear least squares was implemented with scipy.optimize.curve_fit using the Levenberg-Marquardt algorithm and a maximum of 5000 function evaluations. Parameter bounds were set as follows: IVIM: f 0–1, D* 0.005–0.5 mm²/s, D 0.0001–0.003 mm²/s; SEM: DDC 1 × 10⁻⁶–0.01 mm²/s, α 0.1–1.5; FROC: D 1 × 10⁻⁶–0.01 mm²/s, β 0.1–1.5, μ 1–20 μm; CTRW: D 1 × 10⁻⁶–0.01 mm²/s, α 0.1–1.5, β 0.1–3.0. All signals were normalized to the mean b0 signal, and b-values <20 s/mm² were treated as b0. A slice-wise 3×3 mean filter was applied before fitting to reduce high-b-value noise.

Regarding the FROC model, the spatial-scale parameter μ was fitted rather than fixed. A lower bound of μ ≥ 1.0 μm was deliberately imposed. In initial preliminary experiments, a less restrictive lower bound (e.g., μ ≥ 0.01) led to severe parameter coupling and overfitting. Constraining μ ≥ 1.0 μm prevents the algorithm from artificially compensating for high-b-value noise, thereby ensuring robust estimation of D and β. When the true biological μ is smaller than 1.0 μm, the algorithm defaults to this boundary to prevent numerical instability. Consequently, the 10th percentile value of exactly 1.000 reflects this imposed methodological constraint rather than an artifact, indicating regions of extreme diffusion restriction.

The code computed and saved R², but no R² threshold was applied to exclude low-quality fits. Failed fits were assigned a value of 0, and NaN, negative, and some out-of-bound values were also set to 0. This handling may introduce bias and is acknowledged in the Discussion.

Internal quality control analysis showed that the mean R² for all models exceeded 0.88, and the proportion of poorly fitted voxels (defined as R² < 0.8) was less than 5%. Model fitting quality was evaluated using the coefficient of determination (R²). Voxels with R² < 0.8 were defined as poorly fitted.

The specific b-value scheme (including the 1700 and 1800 s/mm² spacing) was intentionally designed to optimize the sampling of the signal decay curve. The dense sampling in this specific high-b-value range was strategically chosen to capture the curvature and inflection points of the non-Gaussian diffusion signal, which is critical for accurately fitting the fractional-order β in the FROC model and estimating kurtosis in DKI.

**1.2 Regression equations:**

(1) M-clinical:

$$\text{logit}\left( P \right)=\beta_{0}+\beta_{1}\times\text{Age}+\beta_{2}\times\text{Pathological}\text{ }\text{t}\text{ype}+\beta_{3}\times\text{MRI-based local stage}$$

The parameter β represents regression coefficient. Age is expressed in years, Pathological type is coded as 1 for NEAC and 0 for EAC, and MRI-based local stage is coded as 1 for tumor extending beyond the corpus and 0 for tumor confined to the corpus.

(2) M-radiomics:

$$\text{logit}\left( P \right)=\beta_{0}+\beta_{1}\times\text{DKI}\text{\_}\text{K}\text{\_}\text{energy}+\beta_{2}\times\text{FROC}\text{\_}\mu\text{\_}\text{10th}\text{ }\text{percentile}$$

The parameter β represents regression coefficient. DKI_K_energy is the energy of the DKI K parameter, and FROC_μ_10th percentile is the 10th percentile of the FROC μ parameter.

(3) M-combined:

$$\text{logit}\left( P \right)=\beta_{0}+\beta_{1}\times\text{DKI}\text{\_}\text{K}\text{\_}\text{energy}+\beta_{2}\times\text{A}\text{ge}+\beta_{3}\times\text{Pathological}\text{ }\text{type}$$

The parameter β represents regression coefficient. DKI_K_energy, Age, and Pathological type are defined as above.

For all three models, the predicted probability of LNM was calculated as:

$$P=\frac{1}{1+e^{-\text{logit}\left( P \right)}}$$

**Supplementary Table 1** Interobserver agreement of histogram parameters using intraclass correlation coefficient (ICC).

| Parameters |  | 10th | 90th | Max | Min | Range | Variance | Kurtosis | RMS | Energy | Skewness | MAD | Mean | IQR | rMAD | Median | Total_E |
| --- | --- | --- | --- | --- | --- | --- | --- | --- | --- | --- | --- | --- | --- | --- | --- | --- | --- |
| IVIM_Df | ICC | 0.958 | 0.789 | 0.814 | 0.875 | 0.815 | 0.924 | 0.781 | 0.923 | 0.979 | 0.782 | 0.923 | 0.940 | 0.982 | 0.923 | 0.993 | 0.978 |
|  | 95%CI | 0.934-0.973 | 0.684-0.862 | 0.719-0.879 | 0.808-0.920 | 0.720-0.880 | 0.881-0.951 | 0.651-0.862 | 0.880-0.951 | 0.966-0.986 | 0.654-0.863 | 0.880-0.951 | 0.906-0.962 | 0.970-0.989 | 0.881-0.951 | 0.988-0.995 | 0.965-0.986 |
|  | *p* | <0.001 | <0.001 | <0.001 | <0.001 | <0.001 | <0.001 | <0.001 | <0.001 | <0.001 | <0.001 | <0.001 | <0.001 | <0.001 | <0.001 | <0.001 | <0.001 |
| IVIM_D | ICC | 0.979 | 0.913 | 0.873 | 0.886 | 0.877 | 0.918 | 0.932 | 0.942 | 0.976 | 0.870 | 0.880 | 0.949 | 0.770 | 0.808 | 0.937 | 0.974 |
|  | 95%CI | 0.967-0.987 | 0.865-0.945 | 0.805-0.918 | 0.825-0.927 | 0.809-0.921 | 0.872-0.947 | 0.895-0.957 | 0.909-0.963 | 0.962-0.985 | 0.800-0.916 | 0.815-0.923 | 0.919-0.968 | 0.658-0.849 | 0.711-0.875 | 0.901-0.960 | 0.960-0.984 |
|  | *p* | <0.001 | <0.001 | <0.001 | <0.001 | <0.001 | <0.001 | <0.001 | <0.001 | <0.001 | <0.001 | <0.001 | <0.001 | <0.001 | <0.001 | <0.001 | <0.001 |
| IVIM_f | ICC | 0.924 | 0.929 | 0.818 | 0.861 | 0.848 | 0.928 | 0.799 | 0.949 | 0.970 | 0.905 | 0.952 | 0.951 | 0.943 | 0.955 | 0.946 | 0.968 |
|  | 95%CI | 0.882-0.952 | 0.890-0.955 | 0.725-0.881 | 0.788-0.861 | 0.769-0.902 | 0.888-0.954 | 0.699-0.869 | 0.919-0.967 | 0.952-0.981 | 0.853-0.939 | 0.925-0.970 | 0.923-0.969 | 0.910-0.964 | 0.930-0.972 | 0.915-0.966 | 0.949-0.980 |
|  | *p* | <0.001 | <0.001 | <0.001 | <0.001 | <0.001 | <0.001 | <0.001 | <0.001 | <0.001 | <0.001 | <0.001 | <0.001 | <0.001 | <0.001 | <0.001 | <0.001 |
| DKI_K | ICC | 0.940 | 0.964 | 0.727 | 0.838 | 0.749 | 0.967 | 0.815 | 0.941 | 0.967 | 0.796 | 0.942 | 0.951 | 0.916 | 0.915 | 0.903 | 0.965 |
|  | 95%CI | 0.906-0.962 | 0.943-0.977 | 0.599-0.819 | 0.755-0.895 | 0.629-0.834 | 0.947-0.979 | 0.721-0.880 | 0.908-0.963 | 0.948-0.979 | 0.675-0.871 | 0.910-0.963 | 0.923-0.969 | 0.870-0.947 | 0.869-0.946 | 0.850-0.938 | 0.944-0.978 |
|  | *p* | <0.001 | <0.001 | <0.001 | <0.001 | <0.001 | <0.001 | <0.001 | <0.001 | <0.001 | <0.001 | <0.001 | <0.001 | <0.001 | <0.001 | <0.001 | <0.001 |
| DKI_D | ICC | 0.991 | 0.917 | 0.897 | 0.971 | 0.885 | 0.891 | 0.943 | 0.940 | 0.972 | 0.819 | 0.875 | 0.946 | 0.774 | 0.813 | 0.911 | 0.969 |
|  | 95%CI | 0.986-0.995 | 0.871-0.947 | 0.841-0.934 | 0.954-0.982 | 0.822-0.926 | 0.831-0.930 | 0.911-0.964 | 0.906-0.962 | 0.955-0.982 | 0.711-0.886 | 0.807-0.920 | 0.914-0.966 | 0.663-0.852 | 0.717-0.878 | 0.861-0.943 | 0.952-0.981 |
|  | *p* | <0.001 | <0.001 | <0.001 | <0.001 | <0.001 | <0.001 | <0.001 | <0.001 | <0.001 | <0.001 | <0.001 | <0.001 | <0.001 | <0.001 | <0.001 | <0.001 |
| SEM_DDC | ICC | 0.981 | 0.966 | 0.930 | 0.945 | 0.921 | 0.982 | 0.916 | 0.974 | 0.991 | 0.724 | 0.949 | 0.975 | 0.930 | 0.935 | 0.959 | 0.991 |
|  | 95%CI | 0.981-0.993 | 0.946-0.978 | 0.891-0.955 | 0.914-0.965 | 0.877-0.950 | 0.972-0.989 | 0.870-0.947 | 0.959-0.984 | 0.985-0.994 | 0.560-0.827 | 0.921-0.968 | 0.961-0.984 | 0.891-0.955 | 0.899-0.959 | 0.935-0.974 | 0.985-0.994 |
|  | *p* | <0.001 | <0.001 | <0.001 | <0.001 | <0.001 | <0.001 | <0.001 | <0.001 | <0.001 | <0.001 | <0.001 | <0.001 | <0.001 | <0.001 | <0.001 | <0.001 |
| SEM_α | ICC | 0.958 | 0.976 | 0.893 | 0.896 | 0.901 | 0.957 | 0.873 | 0.965 | 0.967 | 0.757 | 0.964 | 0.966 | 0.921 | 0.922 | 0.967 | 0.965 |
|  | 95%CI | 0.934-0.974 | 0.962-0.985 | 0.835-0.931 | 0.839-0.933 | 0.847-0.937 | 0.933-0.973 | 0.805-0.918 | 0.945-0.978 | 0.948-0.979 | 0.639-0.840 | 0.941-0.977 | 0.946-0.978 | 0.876-0.950 | 0.877-0.951 | 0.948-0.979 | 0.945-0.978 |
|  | *p* | <0.001 | <0.001 | <0.001 | <0.001 | <0.001 | <0.001 | <0.001 | <0.001 | <0.001 | <0.001 | <0.001 | <0.001 | <0.001 | <0.001 | <0.001 | <0.001 |

**Supplementary Table 1 (continued)**

| Parameters |  | 10th | 90th | Max | Min | Range | Variance | Kurtosis | RMS | Energy | Skewness | MAD | Mean | IQR | rMAD | Median | Total_E |
| --- | --- | --- | --- | --- | --- | --- | --- | --- | --- | --- | --- | --- | --- | --- | --- | --- | --- |
| FROC_D | ICC | 0.927 | 0.959 | 0.930 | 0.773 | 0.706 | 0.942 | 0.982 | 0.964 | 0.966 | 0.989 | 0.936 | 0.963 | 0.906 | 0.924 | 0.960 | 0.961 |
|  | 95%CI | 0.88-0.954 | 0.935-0.974 | 0.891-0.955 | 0.662-0.810 | 0.532-0.815 | 0.909-0.963 | 0.972-0.989 | 0.943-0.977 | 0.947-0.979 | 0.983-0.993 | 0.900-0.959 | 0.941-0.977 | 0.855-0.940 | 0.881-0.951 | 0.937-0.975 | 0.939-0.976 |
|  | *p* | <0.001 | <0.001 | <0.001 | <0.001 | <0.001 | <0.001 | <0.001 | <0.001 | <0.001 | <0.001 | <0.001 | <0.001 | <0.001 | <0.001 | <0.001 | <0.001 |
| FROC_β | ICC | 0.977 | 0.975 | 0.853 | 0.950 | 0.850 | 0.951 | 0.771 | 0.968 | 0.965 | 0.868 | 0.957 | 0.969 | 0.925 | 0.923 | 0.967 | 0.963 |
|  | 95%CI | 0.963-0.985 | 0.961-0.985 | 0.776-0.905 | 0.922-0.969 | 0.771-0.903 | 0.923-0.969 | 0.658-0.849 | 0.950-0.980 | 0.946-0.978 | 0.798-0.915 | 0.933-0.973 | 0.951-0.980 | 0.883-0.952 | 0.880-0.951 | 0.948-0.979 | 0.942-0.977 |
|  | *p* | <0.001 | <0.001 | <0.001 | <0.001 | <0.001 | <0.001 | <0.001 | <0.001 | <0.001 | <0.001 | <0.001 | <0.001 | <0.001 | <0.001 | <0.001 | <0.001 |
| FROC_μ | ICC | 0.865 | 0.943 | 0.881 | 0.847 | 0.895 | 0.938 | 0.921 | 0.964 | 0.961 | 0.932 | 0.949 | 0.972 | 0.964 | 0.948 | 0.964 | 0.961 |
|  | 95%CI | 0.794-0.913 | 0.911-0.964 | 0.817-0.924 | 0.767-0.901 | 0.838-0.933 | 0.903-0.961 | 0.877-0.949 | 0.943-0.977 | 0.939-0.976 | 0.893-0.957 | 0.919-0.968 | 0.955-0.982 | 0.944-0.977 | 0.918-0.967 | 0.944-0.977 | 0.938-0.975 |
|  | *p* | <0.001 | <0.001 | <0.001 | <0.001 | <0.001 | <0.001 | <0.001 | <0.001 | <0.001 | <0.001 | <0.001 | <0.001 | <0.001 | <0.001 | <0.001 | <0.001 |
| CTRW_D | ICC | 0.988 | 0.966 | 0.930 | 0.947 | 0.921 | 0.982 | 0.898 | 0.974 | 0.991 | 0.783 | 0.949 | 0.975 | 0.930 | 0.935 | 0.959 | 0.991 |
|  | 95%CI | 0.981-0.992 | 0.946-0.978 | 0.890-0.955 | 0.961-0.966 | 0.876-0.949 | 0.972-0.989 | 0.834-0.937 | 0.959-0.984 | 0.985-0.994 | 0.658-0.863 | 0.921-0.968 | 0.961-0.984 | 0.891-0.955 | 0.899-0.959 | 0.935-0.974 | 0.985-0.994 |
|  | *p* | <0.001 | <0.001 | <0.001 | <0.001 | <0.001 | <0.001 | <0.001 | <0.001 | <0.001 | <0.001 | <0.001 | <0.001 | <0.001 | <0.001 | <0.001 | <0.001 |
| CTRW_α | ICC | 0.780 | 0.872 | 0.831 | 0.740 | 0.825 | 0.936 | 0.947 | 0.937 | 0.966 | 0.832 | 0.944 | 0.937 | 0.758 | 0.938 | 0.971 | 0.964 |
|  | 95%CI | 0.670-0.856 | 0.804-0.918 | 0.744-0.891 | 0.585-0.837 | 0.736-0.887 | 0.897-0.960 | 0.917-0.967 | 0.901-0.960 | 0.946-0.978 | 0.745-0.891 | 0.911-0.965 | 0.901-0.960 | 0.641-0.841 | 0.938-0.961 | 0.954-0.982 | 0.943-0.977 |
|  | *p* | <0.001 | <0.001 | <0.001 | <0.001 | <0.001 | <0.001 | <0.001 | <0.001 | <0.001 | <0.001 | <0.001 | <0.001 | <0.001 | <0.001 | <0.001 | <0.001 |
| CTRW_β | ICC | 0.958 | 0.893 | 0.810 | 0.896 | 0.835 | 0.746 | 0.907 | 0.936 | 0.958 | 0.718 | 0.908 | 0.948 | 0.921 | 0.915 | 0.967 | 0.956 |
|  | 95%CI | 0.934-0.974 | 0.835-0.931 | 0.714-0.876 | 0.839-0.933 | 0.750-0.893 | 0.624-0.832 | 0.856-0.941 | 0.900-0.959 | 0.934-0.974 | 0.586-0.813 | 0.856-0.941 | 0.919-0.967 | 0.876-0.950 | 0.867-0.946 | 0.948-0.979 | 0.931-0.972 |
|  | *p* | <0.001 | <0.001 | <0.001 | <0.001 | <0.001 | <0.001 | <0.001 | <0.001 | <0.001 | <0.001 | <0.001 | <0.001 | <0.001 | <0.001 | <0.001 | <0.001 |

Intraclass correlation coefficients (ICC), 95% confidence intervals (CI), and p-values are presented for each parameter across different models. 10th = 10th percentile; 90th = 90th percentile; Max = maximum; Min = minimum; RMS = root mean squared; MAD = mean absolute deviation; IQR = interquartile range; rMAD = robust mean absolute deviation; Total_E = total energy.

**Supplementary Table 2** Inter-reader agreement for MRI-based local stage assessment.

| Variable | Reader A (n) | Reader B (n) | Cohen's Kappa | 95 % CI | *P* |
| --- | --- | --- | --- | --- | --- |
| MRI-based local stage |  |  | 0.890 | 0.767-1.000 | < 0.001 |
| stage I | 38 | 42 |  |  |  |
| stage II/III | 35 | 31 |  |  |  |

Cohen's Kappa was used to evaluate inter-reader agreement for MRI-based local stage.

**Supplementary Table 3** Collinearity diagnostics of independent variables in the three logistic regression models.

| Model and Variable | TOL | VIF |
| --- | --- | --- |
| M-clinical |  |  |
| Age | 0.941 | 1.063 |
| Pathological type | 0.929 | 1.076 |
| MRI-based local stage | 0.984 | 1.017 |
| M-radiomics |  |  |
| DKI_K_energy | 0.978 | 1.022 |
| FROC_μ_10th percentile* | 0.978 | 1.022 |
| M-combined |  |  |
| DKI_K_energy | 0.984 | 1.016 |
| Age | 0.941 | 1.062 |
| Pathological type | 0.927 | 1.079 |

TOL, tolerance; VIF, variance inflation factor. MRI-based local stage refers to the preoperative MRI-defined extent of the primary tumor, excluding LNM status. DKI, diffusion kurtosis imaging; FROC, fractional-order calculus. *: ×10⁻³ mm.

**Supplementary Table 4** Bootstrap stability assessment of intercept estimates for the three models (5000 resamples).

| Model | B | Bias | SE | 95 % CI | *P* |
| --- | --- | --- | --- | --- | --- |
| M-clinical | 5.604 | 0.462 | 1.679 | 3.513 - 10.003 | <0.001 |
| M-radiomics | 5.886 | 0.763 | 2.273 | 3.553 - 12.017 | <0.001 |
| M-combined | 6.248 | 0.644 | 5.600 | 4.381 - 10.606 | <0.001 |

Bootstrap resampling was performed with 5000 replicates. B represents the estimated intercept (constant term) of the logistic regression model derived from the original sample. Bias is the difference between the bootstrap mean estimate and the original sample estimate. Standard error is the bootstrap standard error. 95% CI denotes the bias-corrected and accelerated bootstrap confidence interval.

**Supplementary Table 5** Multivariable logistic regression analysis for LNM assessment in the clinical, radiomics, and combined models.

| Model and Variable | β | S.E. | Wald | OR | 95% CI | *P* |
| --- | --- | --- | --- | --- | --- | --- |
| M-clinical |  |  |  |  |  |  |
| Age | 0.081 | 0.039 | 4.447 | 1.085 | 1.006-1.170 | 0.035 |
| Pathological type | -2.241 | 0.788 | 8.092 | 0.106 | 0.023-0.498 | 0.004 |
| MRI-local FIGO stage | 0.983 | 0.429 | 5.258 | 2.673 | 1.153-6.195 | 0.022 |
| Constant | -6.046 | 2.629 | 5.288 | 0.002 |  | 0.021 |
| M-radiomics |  |  |  |  |  |  |
| DKI_K_energy | 0.001 | 0.001 | 11.221 | 1.001 | 1.001-1.002 | ＜0.001 |
| FROC_μ_10th percentile* | 4.591 | 2.229 | 4.240 | 98.560 | 1.247-7787.380 | 0.039 |
| Constant | -5.967 | 2.055 | 8.431 | 0.003 |  | 0.004 |
| M-combined |  |  |  |  |  |  |
| DKI_K_energy | 0.002 | 0.001 | 13.854 | 1.002 | 1.001-1.003 | ＜0.001 |
| Age | 0.151 | 0.055 | 7.658 | 1.163 | 1.045-1.294 | 0.006 |
| Pathological type | -3.026 | 0.980 | 9.534 | 0.048 | 0.007-0.331 | 0.002 |
| Constant | -9.851 | 3.520 | 7.833 | 0.001 |  | 0.005 |

β, regression coefficient; S.E., standard error; Wald, Wald chi-square statistic; OR, odds ratio; 95% CI, 95% confidence interval.

**Supplementary Table 6** STROBE statement checklist of items for observational studies.

| Item | Recommendation | Section |
| --- | --- | --- |
| 1 | (a) Indicate the study’s design with a commonly used term in the title or the abstract; (b) Provide in the abstract an informative and balanced summary of what was done and what was found | Abstract |
| 2 | Explain the scientific background and rationale for the investigation being reported | Introduction |
| 3 | State specific objectives, including any prespecified hypotheses | Introduction |
| 4 | Present key elements of study design early in the paper | Methods 2.1 |
| 5 | Describe the setting, locations, and relevant dates, including periods of recruitment, exposure, follow-up, and data collection | Methods 2.1 |
| 6 | (a) Cohort study—Give the eligibility criteria, and the sources and methods of selection of participants. Describe methods of follow-up. Case-control study—Give the eligibility criteria, and the sources and methods of case ascertainment and control selection. Cross-sectional study—Give the eligibility criteria, and the sources and methods of selection of participants; (b) For matched studies, give matching criteria and number of exposed and unexposed | Methods 2.1, Figure 1 |
| 7 | Clearly define all outcomes, exposures, predictors, potential confounders, and effect modifiers. Give diagnostic criteria, if applicable | Methods 2.2, Methods 2.5 |
| 8 | For each variable of interest, give sources of data and details of methods of assessment (measurement). Describe comparability of assessment methods if there is more than one group | Methods 2.2, Methods 2.3, Methods 2.4 |
| 9 | Describe any efforts to address potential sources of bias | Methods 2.5, Methods 2.6 |
| 11 | Explain how quantitative variables were handled in the analyses. If applicable, describe which groupings were chosen and why | Methods 2.6 |
| 12 | (a) Describe all statistical methods, including those used to control for confounding; (b) Describe any methods used to examine subgroups and interactions; (c) Explain how missing data were addressed; (d) If applicable, explain how loss to follow-up was addressed; (e) Describe any sensitivity analyses | Methods 2.6 |
| 13 | (a) Report numbers of individuals at each stage of study; (b) Give reasons for non-participation at each stage; (c) Consider use of a flow diagram | Results 3.1, Figure 1 |
| 14 | (a) Give characteristics of study participants and information on exposures and potential confounders; (b) Indicate number of participants with missing data for each variable of interest; (c) Summarise follow-up time | Results 3.2, Results 3.3, Results 3.4 |
| 15 | Report numbers of outcome events or summary measures | Results 3.1, Table 1 |
| 16 | (a) Give unadjusted estimates and, if applicable, confounder-adjusted estimates and their precision (e.g., 95% confidence interval); (b) Report category boundaries when continuous variables were categorized; (c) If relevant, consider translating estimates of relative risk into absolute risk for a meaningful time period | Results 3.3, Results 3.4, Results 3.5 |
| 17 | Report other analyses done—e.g., analyses of subgroups and interactions, and sensitivity analyses | Results 3.4 |
| 18 | Summarise key results with reference to study objectives | Discussion |
| 19 | Discuss limitations of the study, taking into account sources of potential bias or imprecision. Discuss both direction and magnitude of any potential bias | Limitations |
| 20 | Give a cautious overall interpretation of results considering objectives, limitations, multiplicity of analyses, results from similar studies, and other relevant evidence | Discussion, Conclusion |
| 21 | Discuss the generalisability (external validity) of the study results | Discussion |
| 22 | Give the source of funding and the role of the funders for the present study and, if applicable, for the original study on which the present article is based | Funding |

This checklist follows the STROBE guidelines.

**Supplementary Table 7** Radiomics Quality Score (RQS) assessment.

| No. | RQS Item | Score Criteria / Explanation | Score |
| --- | --- | --- | --- |
| 1 | Image protocol quality | Detailed MRI acquisition parameters are fully documented (TR, TE, FOV, matrix, slice thickness, b values). However, the full protocol is not publicly accessible. | 1 |
| 2 | Multiple segmentations | Two radiologists independently delineated the VOI. Inter observer agreement was evaluated using the intraclass correlation coefficient (ICC 0.568 to 0.993). | 1 |
| 3 | Phantom study | No phantom study was performed to assess the impact of scanner variability on feature robustness. | 0 |
| 4 | Multiple time points | No test retest or multiple time point scans were performed to evaluate temporal variability. | 0 |
| 5 | Feature reduction | Univariate Mann Whitney U test, LASSO regression with 10 fold cross validation, and collinearity diagnostics (TOL, VIF) were applied. | 3 |
| 6 | Multivariable analysis | The combined model incorporated non radiomic features including age and pathological type. | 1 |
| 7 | Biological correlation | Biological meanings of DKI K energy and FROC mu were discussed in relation to tumor microstructural heterogeneity and proliferation. | 1 |
| 8 | Cut off analysis | Optimal cut off values were determined using ROC curves and the Youden index to stratify risk groups. | 1 |
| 9 | Discrimination statistics | AUC values with 95% confidence intervals and P values were reported. Bootstrap internal validation with 5000 resamples was performed. | 2 |
| 10 | Calibration statistics | Calibration curves and Brier scores were reported. Bootstrap resampling supported the stability of the calibration. | 2 |
| 11 | Prospective design | This study prospectively collected patient data as a single center observational cohort. It is not a clinical trial designed to prospectively validate radiomic features. | 0 |
| 12 | Validation | Internal validation was performed using Bootstrap resampling with 5000 replicates. No external validation was conducted. | 2 |
| 13 | Comparison to gold standard | Model performance was compared against postoperative pathological lymph node status as the gold standard. | 2 |
| 14 | Potential clinical utility | Decision curve analysis demonstrated higher net benefit. The model may aid preoperative risk stratification and individualized treatment decisions. | 2 |
| 15 | Cost effectiveness analysis | No formal cost effectiveness analysis was performed. | 0 |
| 16 | Open science and data | The original data are available upon reasonable request to the corresponding author, but no public scans, segmentations, or code links are provided. | 0 |
| Total |  | Total Radiomics Quality Score (RQS) for this study. | 18 |

**Supplementary Figure 1**

Distribution of the three LASSO-selected histogram features according to LNM status. DKI, diffusion kurtosis imaging; FROC, fractional-order calculus; LNM, lymph node metastasis.
